# Disseminated herpes zoster in adults: a single-center retrospective study on clinical spectrum and risk factors for adverse outcomes

**DOI:** 10.3389/fmed.2026.1760499

**Published:** 2026-02-04

**Authors:** Jia-luo Cai, Jin-guo Fan, An-rui Yi

**Affiliations:** Department of Rehabilitation, University-Town Hospital of Chongqing Medical University, Chongqing, China

**Keywords:** clinical, DHZ, disseminated herpes zoster, management strategies, varicella-zoster virus

## Abstract

**Background:**

Disseminated herpes zoster (DHZ) is defined by the involvement of more than two non-contiguous dermatomes or the presence of at least 20 lesions beyond the primary affected dermatome. The varicella-zoster virus (VZV) can lead to disseminated infection when the host’s immune response is insufficient to halt cell-associated viremia. This study aimed to summarize the clinical characteristics and potential risk factors associated with DHZ and to identify and assess factors related to prognosis to enhance the prevention and management strategies for DHZ patients.

**Methods:**

The demographic information of DHZ patients who presented to our department between January 2019 and January 2025 was retrospectively collected. Patients with a confirmed diagnosis of DHZ were monitored to identify morbidity risk factors and the incidence of postherpetic neuralgia.

**Results:**

A total of 109 patients were diagnosed with DHZ, with a male predominance (65 patients, 59.63%) and 44 female patients (40.37%). The mean age of the patients was 68.6 years. Advanced age (≥65 years) was the most prevalent demographic risk factor. In the assessment of prognostic outcomes, concomitant conditions including hypertension, diabetes, and cancer, along with severe pain (VAS score 7–10), were significantly associated with an increased risk of postherpetic neuralgia in a univariate analysis. Furthermore, a multivariable logistic regression analysis identified cancer as an independent risk factor for prolonged disease duration (OR = 4.271, *p* = 0.002).

**Conclusion:**

This study identified advanced age, cardiovascular disease, and chronic inflammation as independent risk factors for DHZ, highlighting potential target groups for vaccination. Moreover, factors such as hypertension, diabetes, and severe initial pain were associated with postherpetic neuralgia (PHN) in a univariate analysis, suggesting avenues for early attention. These findings highlight the importance of early recognition, aggressive management, and, most importantly, targeted preventive vaccination with the non-live recombinant vaccine in high-risk populations, including cancer patients, to reduce the substantial morbidity associated with disseminated herpes zoster.

## Introduction

Herpes zoster (HZ), caused by the reactivation of the latent varicella-zoster virus (VZV) within the sensory ganglia and dorsal roots (1, 2), is characterized by centrally distributed blistering skin lesions that are rich in infectious virus, predominantly affecting older adults (3). Disseminated herpes zoster (DHZ) represents a severe complication of VZV infection (4). It is widely acknowledged that immunocompromised status is a primary risk factor for DHZ, leading to specific symptoms. Immunocompromised status is associated with increased morbidity and reduced quality of life (5). These individuals face an increased risk of diagnostic delay and treatment challenges for DHZ. Therefore, it is crucial to identify the clinical and demographic characteristics of these patients.

DHZ is characterized by an eruption of at least 20 widespread vesiculobullous lesions beyond the primary and adjacent dermatomes (4). Atypical HZ, which occurs in 10–40% of immunocompromised individuals, may involve visceral organs (6–8). Although rare, DHZ outbreaks can be extremely severe, potentially affecting the liver, lungs, and even the central nervous system. Consequently, a deeper understanding of the disease is essential. Current research on DHZ is predominantly limited to case analyses, with few multi-sample studies reported to date. Several studies indicate that the HZ vaccine can prevent both HZ and postherpetic neuralgia in older adults (1, 9), offering a preventive strategy for high-risk populations, which is also applicable to DHZ.

Our study has focused on clinical characteristics and prognostic factors of DHZ based on sex, age, prodromal symptoms, morbidity, and prognosis. By analyzing patients diagnosed with DHZ, this study provides a better understanding of disease prevention and treatment and could also help to increase awareness among healthcare professionals and guide better prevention and patient care strategies. We evaluated the disease burden caused by DHZ, including the duration of treatment and cost of treatment among the patients, which helps to understand the disease better.

## Methods

This study was designed as a retrospective cohort study. Comprehensive anamnesis was collected from patients, including age, sex, season of morbidity, disease duration, clinical characteristics, history of herpes zoster vaccination, medication history, including recent use of systemic corticosteroids, and comorbidities. Patients with postherpetic neuralgia were compared with those without it concerning their DHZ-related morbidity during the follow-up period.

### Study population and endpoints

This single-center retrospective study included patients diagnosed with DHZ who were admitted to the Department of Rehabilitation at the University-Town Hospital of Chongqing Medical University between January 2019 and May 2025. Chongqing is a major municipality in southwestern China, with a population of over 30 million. All diagnoses were confirmed by dermatologists. Relevant demographic and clinical data were extracted from the hospital’s electronic medical record system. The study collected the clinical and demographic characteristics of DHZ patients. The primary outcomes were the characteristics of DHZ and the prevalence of comorbidities, which included hypertension, diabetes mellitus, dyslipidemia, and coronary artery disease. The secondary outcome was the incidence of postherpetic neuralgia (PHN), defined as pain persisting for more than 90 days after rash healing. The collected laboratory parameters included white blood cell count (WBC), lymphocyte count, platelet count, alanine aminotransferase (ALT), and creatinine.

### Statistical analysis

Descriptive statistics were presented for the entire study population and stratified according to DHZ-exposure status. Continuous variables were expressed as means ± standard deviation (SD), medians, and minimum/maximum values. Categorical variables were presented as frequencies and percentages of the available cases, and comparisons were performed using the chi-squared test. Continuous variables were compared using the independent samples *t*-test. A two-tailed *p*-value of <0.05 was considered the threshold for statistical significance testing. SPSS 29.0 was used for all data analyses. Univariate analyses of odds ratios (ORs) for each variable and incidence rates were conducted using ordinal logistic regression analyses for each confounder in the database. Multivariable models were constructed using variables that were significant (*p* regression), and the results are shown as ORs [95% confidence intervals (CIs)]. The assumption of parallel lines was assessed by means of the likelihood ratio test. Multicollinearity was assessed by checking the variance inflation factors in the multiple regression model.

## Results

### Demographic characteristics and underlying diseases of DHZ patients

This study included a total of 109 patients with DHZ. The median age at diagnosis was 72 years (mean ± standard deviation: 68.60 ± 12.41 years), with an age range of 14–96 years. Among them, 72 patients (66.1%) were aged ≥65 years, while 37 patients (33.9%) were aged <65 years. There were 65 male (59.6%) and 44 female patients (40.4%), with a male-to-female ratio of approximately 1.48:1. In terms of seasonal distribution, spring had the highest proportion of cases (46 patients, 42.2%), followed by summer (29 patients, 26.6%), autumn (23 patients, 21.1%), and winter (11 patients, 10%). Regarding underlying comorbidities among the 109 patients, hypertension was the most common (68 patients, 62.4%), followed by diabetes mellitus (43 patients, 39.4%) and malignancy (21 patients, 19.3%). Other comorbidities included dyslipidemia (34 patients, 31.2%), coronary artery disease (33 patients, 30.3%), and chronic kidney disease (24 patients, 22.0%). Regarding zoster vaccination history, 59 (54.1%) patients reported yes, 38 (34.9%) reported no, and 12 (11.0%) reported did not know. The specific type of zoster vaccine—live-attenuated versus recombinant—was not consistently documented in the medical records. The distribution of skin lesions and baseline characteristics of these patients are detailed in Table 1.

**Table 1:** Clinical data about the 109 patients with disseminated herpes zoster.

Variable	DHZ (*N* = 109)
Age, years	72 (IQR: 61–79)
≥65 years, *n* (%)	72 (66.1%)
<65 years, *n* (%)	37 (33.9%)
Male, *n* (%)	65 (59.6%)
Female, *n* (%)	44 (40.4%)
Male-to-female ratio	1.48:1
History of zoster vaccination, *n* (%)	
Yes	59 (54.1%)
No	38 (34.9%)
Unknown	12(11.0%)
Season of onset, *n* (%)	
Spring	46 (42.2%)
Summer	29 (26.6%)
Autumn	23 (21.1%)
Winter	11 (10.0%)
Lesion distribution, *n* (%)	
Chest and back	78 (71.6%)
Waist and abdomen	46 (42.2%)
Head and face	39 (35.8%)
Limbs	38 (34.9%)
Neck	12 (11.0%)

Age is presented as median (interquartile range, IQR), i.e., 72 (61–79) years. Categorical variables are presented as *n* (%).

### Temporal distribution of DHZ cases

The first confirmed case of COVID-19 at our hospital was diagnosed on 11 March 2022. The annual distribution of DHZ cases from 2019 to January 2025 was as follows: 2019 (*n* = 12), 2020 (*n* = 17), 2021 (*n* = 20), 2022 (*n* = 28), 2023 (*n* = 21), 2024 (*n* = 10), and January 2025 (*n* = 1). Due to the retrospective design of this study and the absence of systematic SARS-CoV-2 testing for all inpatients throughout the study, particularly prior to 2022, we cannot reliably determine the number or proportion of DHZ patients who had a concurrent or recent COVID-19 infection for each calendar year.

### Clinical presentation of the DHZ patients

Regarding the temporal relationship between pain and blister onset, approximately one-third (35.78%) of patients reported the synchronous appearance of both symptoms. In half of the cases (50.46%), pain preceded the development of skin lesions, while blisters appeared first in 13.76% of patients. The blister diameters were predominantly small, with 47.71% measuring less than 3 mm and 37.61% measuring between 3 and 5 mm; larger blisters (>5 mm) were observed in 14.68% of cases. The median number of involved dermatomes was 3 (range: 2–6). The rash most frequently involved the chest and back (71.6%), followed by the waist and abdomen (42.2%), the limbs (34.9%), the head and face (35.8%), and the neck (11.0%). The clinical characteristics of the cohort are detailed in Table 2.

**Table 2:** Clinical presentation of the 109 patients with disseminated herpes zoster.

Characteristics	Total *N* = 109 (100%)
The diameter of the blister, *N* (%)	
<3 mm	52 (47.71%)
3–5 mm	41 (37.61%)
>5 mm	16 (14.68%)
Herpes site, *N* (%)	
Head and face	39 (35.8%)
Neck	12 (11.0%)
Chest and back	78 (71.6%)
Waist and abdomen	46 (42.2%)
Limbs	38 (34.9%)
The order of pain and blister	
Pain as the initial symptom, *N* (%)	55 (50.46%)
Blister as the initial symptom, *N* (%)	15 (13.76%)
Synchronism, *N* (%)	39 (35.78%)
Lesion	
Erosion, *N* (%)	45 (41.28%)
Ulceration, *N* (%)	28 (25.69%)
Pustules, *N* (%)	11 (10.09%)
Bloody vesicle, *N* (%)	15 (13.76%)
Swell, *N* (%)	22 (20.18%)

### Factors associated with prolonged disease duration: a univariate analysis

Based on the analysis presented in Table 3, among the 109 DHZ patients in our cohort, 31 (28.4%) experienced a prolonged disease duration (≥7 days). The presence of cancer was identified as a significant factor associated with extended illness (*p* = 0.007). In contrast, other common variables, including age, sex, comorbidities such as hypertension and diabetes, and routine laboratory parameters, did not show statistically significant differences between patients with and those without prolonged duration. The detailed comparative analysis of these factors is summarized in Table 3.

**Table 3:** Factors associated with prognosis for disseminated herpes zoster.

Variable	Prolonged duration (≥7 days) (*N* = 31)	Non-prolonged duration (<7 days) (*N* = 78)	*χ*^2^/*t*	*p*-value
Age (≥65 vs. <65 years)				
<65 years	10 (32.3%)	40 (51.3%)	3.207	0.073
≥65 years	21 (67.7%)	38 (48.7%)		
Sex (male vs. female)				
Male	20 (64.5%)	45 (57.7%)	0.425	0.514
Female	11 (35.5%)	33 (42.3%)		
Comorbidity				
Hypertension	20 (64.5%)	48 (61.5%)	0.085	0.770
Diabetes	14 (45.2%)	29 (37.2%)	0.595	0.441
Dyslipidemia	8 (25.8%)	26 (33.3%)	0.595	0.441
Coronary disease	10 (32.3%)	23 (29.5%)	0.080	0.777
Chronic kidney disease	6 (19.4%)	18 (23.1%)	0.175	0.676
Cancer	11 (35.5%)	10 (12.8%)	7.404	0.007*
COPD	5 (16.1%)	6 (7.7%)	1.788	0.181
Cerebrovascular disease	7 (22.6%)	11 (14.1%)	1.142	0.285
Corticosteroid use	23 (74.2%)	15 (19.2%)	29.51	<0.001*
Laboratory findings (mean ± SD)				
WBC (×10^9^/L)	5.98 ± 2.67	5.70 ± 2.16	0.554	0.581
Lymphocyte (×10^9^/L)	1.49 ± 0.81	1.65 ± 0.91	−0.835	0.406
Platelet (×10^9^/L)	172.7 ± 63.5	172.4 ± 52.0	0.023	0.982
ALT (U/L)	24.6 ± 33.8	22.3 ± 26.6	0.355	0.723
Creatinine (μmol/L)	77.9 ± 22.3	72.9 ± 20.0	1.118	0.266

Prolonged duration was defined as a disease course lasting ≥7 days. Categorical variables are expressed as *n* (%) and were compared using the chi-squared test (*X*^2^ values shown). Continuous variables are expressed as mean ± standard deviation and were compared using the independent samples *t*-test (*t* values shown). COPD, chronic obstructive pulmonary disease; WBC, white blood cell count; ALT, alanine aminotransferase.

^*^p < 0.05 was considered statistically significant. ^**^p < 0.001 denotes a highly significant association. Percentages are calculated based on the column total for each group.

### Independent predictors of prolonged disease course in DHZ

Based on the multivariable logistic regression analysis presented in Table 4, cancer was identified as an independent and significant predictor of prolonged disease duration (≥7 days) in patients with DHZ (OR = 4.271, 95% CI: 1.688–10.807, *p* = 0.002). In contrast, other variables, including age, sex, hypertension, diabetes, coronary disease, and markers of immune status such as lymphopenia, were not independently associated with prolonged illness in the adjusted model. These findings indicate that, among the factors analyzed, an underlying malignancy is the strongest modifiable risk factor associated with a protracted clinical course in DHZ. Systemic corticosteroid use was documented in 38 of 109 patients (34.9%). This use was significantly more prevalent in patients with a prolonged disease course (23/31, 74.2%) compared to those without a prolonged disease course (15/78, 19.2%) (*χ*^2^ = 29.51, *p* < 0.001, Table 3).

**Table 4:** Multifactorial logistic regression analysis of risk factors for prolonged disease duration (≥7 days) in patients with disseminated herpes zoster.

Variable	*β*	SE	Wald	*p*-value	OR (95% CI)
Age (≥65 vs. <65 years)	0.712	0.418	2.903	0.088	2.038 (0.899–4.621)
Sex (female)	−0.285	0.432	0.435	0.509	0.752 (0.322–1.755)
Hypertension	−0.126	0.422	0.089	0.765	0.882 (0.386–2.014)
Diabetes	0.338	0.445	0.577	0.447	1.402 (0.586–3.354)
Coronary disease	−0.105	0.468	0.050	0.823	0.900 (0.360–2.252)
Cancer	1.452	0.473	9.418	0.002*	4.271 (1.688–10.807)
Chronic kidney disease	−0.225	0.562	0.160	0.689	0.799 (0.265–2.405)
COPD	0.892	0.625	2.038	0.153	2.440 (0.716–8.315)
Cerebrovascular disease	0.524	0.559	0.878	0.349	1.688 (0.564–5.056)
Lymphocyte <1.0 × 10^9^/L	0.401	0.440	0.830	0.362	1.493 (0.630–3.537)

CI, confidence interval. This table presents the results of a multivariable logistic regression analysis examining factors associated with a prolonged disease duration (≥7 days) in DHZ. All independent variables were entered into the model with “No” as the reference category. An odds ratio (OR) of >1 indicates that the variable is a risk factor, while an OR of <1 suggests a potentially protective effect. ^*^p < 0.05 was considered statistically significant.

## Discussion

DHZ represents an atypical manifestation of VZV infection, typically characterized by a vesicular rash comprising more than 20 lesions beyond a single dermatome (4, 10). Due to its relative rarity and atypical presentation, DHZ is frequently subject to diagnostic delay (4). In our study of 109 patients, we analyzed demographic and clinical variables to delineate the profile of this condition. Our cohort had a mean age of 68.6 years and a male predominance (59.6%), indicating that older male patients constitute a key risk group. The disease showed a seasonal predilection, with the highest incidence observed in the spring (42.2%). The observed peak incidence in spring warrants further investigation but may be related to seasonal fluctuations in immune function or to environmental factors that influence viral reactivation. The clinical course imposed a substantial burden, with a median hospitalization duration of 10 days, underscoring the significant impact on patients’ quality of life and healthcare resources. Consequently, enhancing prevention and early diagnosis is crucial to mitigate this burden.

In this cohort, 59 patients (54.1%) had a history of zoster vaccination. This finding indicates that a substantial proportion of DHZ cases in our study represent breakthrough infections. Although the recombinant zoster vaccine (RZV) is highly effective in preventing herpes zoster and its complications in the general older population (11, 12), our data suggest that, for high-risk individuals with profound immunosuppressive risk factors such as malignancy, vaccine-derived protection may be insufficient to completely prevent severe, disseminated disease. This finding underscores that, for these extremely vulnerable patients, heightened clinical vigilance for DHZ must be maintained even in the presence of a vaccination history. Our core finding—that malignancy is an independent risk factor for prolonged disease course—gains further significance in this context, as it identifies the precise subgroup that requires intensified monitoring and management.

A clear understanding of the clinical presentation is vital to prevent misdiagnosis. In our cohort, the rash most commonly involved the trunk (chest/back: 71.6%; waist/abdomen: 42.2%). The predominance of truncal involvement (chest/back and waist/abdomen) in our cohort is consistent with the typical distribution of HZ, although the high percentage of multi-dermatomal spread distinguishes DHZ from its localized counterpart (13). Regarding symptomatology, half of the patients (50.5%) reported pain preceding the blister eruption, while 35.8% experienced synchronous onset. The blister diameters were predominantly small (<5 mm in 85.3% of cases). The progression of DHZ mirrors that of HZ (14), typically beginning with erythematous papules that evolve into vesicles within 24–72 h. In severe or immunocompromised cases, this diffuse rash can lead to serious complications, such as encephalitis, hepatitis, or pneumonitis (15). However, no such visceral complications were observed in the present cohort, which may reflect effective early antiviral intervention or the specific immune status of our patients.

A primary focus of our analysis was to identify factors associated with a prolonged disease course (≥7 days), which serves as a marker of severe or complicated illness. While PHN remains a critical complication, our data allowed for a robust analysis of duration. We identified cancer as an independent and significant predictor of prolonged illness (OR = 4.271, *p* = 0.002). This finding aligns with the established understanding that immunosuppressed states, including malignancy and its treatments, predispose individuals to more severe and protracted VZV reactivations (4, 16, 17). These patients are at greater risk for visceral dissemination and require heightened vigilance.

A notable finding of our study is the strong association between systemic corticosteroid use and a prolonged clinical course of DHZ. Corticosteroids are known to suppress cell-mediated immunity, thereby facilitating VZV reactivation and severe disease (18, 19). In our cohort, corticosteroid use was the factor most strongly associated with prolonged illness in the univariate analysis. This finding underscores corticosteroid therapy as a critical, prevalent, and modifiable iatrogenic risk factor for severe DHZ. For patients requiring such immunosuppression, clinicians should maintain a high index of suspicion for DHZ, initiate early antiviral therapy, and consider prophylactic vaccination with the non-live recombinant zoster vaccine.

Our study period (2019–2025) overlapped with the COVID-19 pandemic, with the first confirmed case at our institution occurring in March 2022. While some reports suggest an association between SARS-CoV-2 infection and herpes zoster (20), the lack of systematic COVID-19 testing data in our cohort precludes an analysis of individual-level co-infection. The observed fluctuations in annual DHZ case numbers may reflect complex interactions between viral epidemiology, healthcare-seeking behavior, and population immunity during this period.

Although the present study was not designed to evaluate long-term cardiovascular outcomes, the high prevalence of hypertension and other cardiovascular comorbidities in our DHZ cohort underscores the need for integrated care. This is particularly relevant given emerging evidence linking HZ to an increased risk of stroke and myocardial infarction (21–23); our multifactorial analysis specifically highlights the clinical context of DHZ. The management of pain, whether acute or chronic (PHN), remains a cornerstone of care. Early antiviral therapy is essential to reduce viral replication and acute symptoms (24). For neuropathic pain, a stratified approach using topical agents (e.g., lidocaine and capsaicin) and systemic medications (e.g., gabapentinoids and tricyclic antidepressants) is recommended, with careful consideration of side effects, particularly in the elderly and those with comorbidities (25).

Given the significant burden of DHZ, particularly in high-risk groups, prevention is paramount. Vaccination is a proven strategy to reduce the incidence of HZ and its complications, including PHN (1, 9, 24). The recombinant zoster vaccine (RZV) is highly effective and recommended for immunocompetent adults aged ≥50 and those aged ≥19 who are or will be immunodeficient (1, 26, 27). Crucially, RZV is a non-live vaccine, making it safe for use in immunocompromised individuals, for whom the live-attenuated vaccine (ZVL) is contraindicated due to the risk of vaccine-related disseminated disease (28). Our findings underscore that patients with cancer—a major risk factor for prolonged DHZ—represent a critical target population for vaccination with RZV to prevent severe outcomes.

This study has several limitations. First, as a single-center retrospective study, selection bias may exist. Second, individual-level data on laboratory-confirmed SARS-CoV-2 infection status were not available for the majority of patients, especially those admitted before 2022. This fundamental data gap prevents any valid assessment of COVID-19 as an acute risk factor for DHZ in our study population. Third, the specific type of zoster vaccine (live-attenuated versus recombinant) received by patients was not consistently documented, thereby limiting our ability to analyze the potential protective effect of different vaccine types.

## Conclusion

In summary, our study of 109 DHZ patients delineates a clinical profile characterized by older age, male sex, and frequent trunk involvement. We identified underlying malignancy as the strongest independent risk factor for a prolonged disease course, reinforcing the association between immunosuppression and severe VZV manifestations. These findings highlight the importance of early recognition, aggressive management, and, most importantly, targeted preventive vaccination with the non-live recombinant vaccine in high-risk populations, including cancer patients, to reduce the substantial morbidity associated with disseminated herpes zoster. Moreover, the potential interaction between COVID-19 and DHZ could not be definitively assessed due to limitations in retrospective testing data; this relationship warrants prospective investigation.

## Data Availability

The original contributions presented in the study are included in the article/supplementary material; further inquiries can be directed to the corresponding author.
